# HDL Cholesterol Efflux and the Complement System Are Linked in Systemic Lupus Erythematosus

**DOI:** 10.3390/jcm12165405

**Published:** 2023-08-20

**Authors:** María García-González, Fuensanta Gómez-Bernal, Juan C. Quevedo-Abeledo, Yolanda Fernández-Cladera, Agustín F. González-Rivero, Raquel López-Mejías, Federico Díaz-González, Miguel Á. González-Gay, Iván Ferraz-Amaro

**Affiliations:** 1 Division of Rheumatology, Hospital Universitario de Canarias, 38320 Tenerife, Spain; margagon23@hotmail.com (M.G.-G.); federico.diaz.gonzalez@gmail.com (F.D.-G.); 2 Division of Central Laboratory, Hospital Universitario de Canarias, 38320 Tenerife, Spain; fuensanta95@gmail.com (F.G.-B.); yolanda.fernandezcladera@gmail.com (Y.F.-C.); afgonriv@gmail.com (A.F.G.-R.); 3 Division of Rheumatology, Hospital Doctor Negrín, 35010 Las Palmas de Gran Canaria, Spain; quevedojcarlos@yahoo.es; 4 Epidemiology, Genetics and Atherosclerosis Research Group on Systemic Inflammatory Diseases, Instituto de Investigación sanitaria Marqués de Valdecilla (IDIVAL), 39011 Santander, Spain; rlopezmejias78@gmail.com; 5 Department of Internal Medicine, University of La Laguna (ULL), 38200 Tenerife, Spain; 6 Division of Rheumatology, IIS-Fundación Jiménez Díaz, 28040 Madrid, Spain; 7 Department of Medicine and Psychiatry, University of Cantabria, 39005 Santander, Spain

**Keywords:** cholesterol efflux capacity, complement system, systemic lupus erythematosus

## Abstract

Cholesterol efflux capacity (CEC), the ability of high-density lipoprotein (HDL) cholesterol to accept cholesterol from macrophages, has been linked to cardiovascular events. Systemic lupus erythematosus (SLE) is characterized by the consumption of complement (C) proteins and has been associated with an increased risk of cardiovascular disease. CEC is reduced in SLE patients compared to controls. In the present work, our objective was to analyze whether the disruption of C influences CEC in patients with SLE. New-generation functional assays of the three pathways of the C system were performed in 207 patients with SLE. Additionally, serum levels of inactive (C1q, C2, C3, C4, and factor D) and activated (C3a) molecules, and regulators (C1-inhibitor and factor H) of C system were measured. CEC, using an in vitro assay, and lipoprotein serum concentrations were assessed. Multivariable linear regression analysis was performed to assess the relationship between C system and CEC. After full multivariable analysis, the alternative C cascade functional test showed a significant and negative relationship with CEC. This was also the case for C2 and C3, in which the associations were found to be positive and statistically significant, after adjustment for covariates. In conclusion, C system and CEC are interconnected in patients with SLE.

## 1. Introduction

Cholesterol efflux capacity (CEC) is the ability of an individual’s high-density lipoproteins (HDL) to promote cholesterol efflux from cholesterol-donor cells, such as macrophages. In other words, CEC measures the process of reverse HDL cholesterol transport, whereby excess cellular cholesterol from peripheral tissues is transported to the liver, where it is excreted [1]. CEC has been inversely associated with the incidence of cardiovascular (CV) events in population-based cohorts [2].

Systemic lupus erythematosus (SLE) is associated with several CV manifestations, of which accelerated atherosclerosis with coronary artery disease is a major cause of morbidity and premature death [3]. In addition to a higher prevalence of traditional risk factors, glucocorticoid therapy, active disease, lupus nephritis, and antiphospholipid antibodies predispose SLE patients to a high risk of CV events [3]; besides, these patients often have an altered lipid profile [4]. They have reduced CEC compared to matched controls regardless of other inflammation-related lipid pattern modifications that occur during the disease [5,6], and this has been associated with the presence of carotid plaques in patients with SLE. However, the exact mechanism that leads to impaired CEC in these patients remains unknown [6].

It has been described that the complement (C) system plays an important role in the pathogenesis of SLE. In this sense, SLE is associated with the activation and consumption of C, which can cause tissue damage. It is known that hereditary C deficiency can cause SLE, and disease processes in SLE result in the development of autoantibodies against certain C proteins [7]. In the present study, we have evaluated, in a large group of patients with SLE, the three pathways of the C system through functional assays and the measurement of the individual components of C belonging to these three pathways. Our objective was to analyze the relationship between this complete characterization of the C system and CEC in patients with SLE.

## 2. Materials and Methods

### 2.1. Study Participants

This cross-sectional study encompassed 207 individuals diagnosed with SLE. All participants were aged 18 years or older, possessed a clinical SLE diagnosis, and satisfied a minimum of 4 classification criteria for the disease as outlined by the American College of Rheumatology (ACR) [8]. Rheumatologists confirmed their SLE diagnoses, and these patients received regular monitoring at rheumatology outpatient clinics. Exclusions from the study involved individuals with a history of chronic liver ailments, cancer, renal failure, ongoing acute or chronic infections, or any other chronic autoimmune condition not directly linked to SLE (excluding conditions such as antiphospholipid syndrome and/or Sjögren’s syndrome associated with SLE). The chosen participants displayed no known medical conditions or drug regimens that might impact lipid levels, and they were not utilizing lipid-lowering drugs apart from statins. The research adhered to the principles outlined in the Declaration of Helsinki. The study’s protocol garnered approval from the Institutional Ethics Committees at Hospital Universitario de Canarias and Hospital Universitario Doctor Negrín (both situated in Spain), and all participants granted informed written consent (approval number 2015_84).

### 2.2. Data Collection

Patients who participated in the study were required to complete a questionnaire regarding their medication usage and also underwent a thorough physical examination. To ensure accuracy, their medical records were carefully reviewed to confirm specific diagnoses and medications. The researchers assessed the disease activity and damage related to SLE using established tools, namely, the Systemic Lupus Erythematosus Disease Activity Index-2000 (SLEDAI-2K) [9] and the Systemic Lupus International Collaborating Clinics/American College of Rheumatology (SLICC/ACR) Damage Index (SDI) [10], respectively. The SLEDAI-2K index was categorized into different levels of disease activity, including none (0 points), mild (1–5 points), moderate (6–10 points), high (11–19 points), and very high activity (>20 points), as previously defined [11]. The disease’s severity was also measured using the Katz index [12].

### 2.3. Lipids and Cholesterol Efflux Assessments

Fasting serum samples were gathered and promptly frozen at −80 °C for subsequent analysis of circulating lipids. Cholesterol, triglyceride, and HDL cholesterol levels underwent measurement through the enzymatic colorimetric assay (Roche). Lipoprotein A and lipoproteins were evaluated via a quantitative immunoturbidimetric assay (Roche). Cholesterol levels ranged from 0.08 to 20.7 mmol/L, with an intra-assay coefficient of variation at 0.3%. Triglyceride levels spanned from 4 to 1000 mg/dL, carrying an intra-assay coefficient of variation of 1.8%. HDL cholesterol levels ranged from 3 to 120 mg/dL, accompanied by an intra-assay coefficient of variation at 0.9%. The atherogenic index was determined by employing the total cholesterol:HDL cholesterol ratio as per the Castelli formula. LDL cholesterol levels were derived using the Friedewald formula. High-sensitivity CRP was assessed using a standard technique.

Macrophage-specific cholesterol efflux capacity (CEC) was evaluated utilizing BODIPY cholesterol, adhering to a previously established procedure [13]. Initially, J774 macrophages were seeded in a 96-well plate at a density of 7 × 10^4^ cells per well. On the following day, the cells were exposed to BODIPY-tagged cholesterol (25 µM; Avanti Polar Lipids), 0.2% BSA, and 2 μg/mL ACAT inhibitor Sandoz 58-035 (Sigma-Aldrich, Darmstadt, Germany) in Roswell Park Memorial Institute (RPMI) medium supplemented with 1% fetal bovine serum (FBS) for a duration of 1 h. Afterward, the cells were rinsed with MEM-HEPES and incubated overnight in serum-free RPMI supplemented with 0.3 mM cAMP, 0.2% BSA, and 2 μg/mL ACAT inhibitor. Apolipoprotein B-depleted plasma was obtained from study participants using polyethylene glycol precipitation. Subsequent to washing the BODIPY cholesterol-labeled cells again with MEM-HEPES, they were subjected to a 4 h incubation with 2.8% apolipoprotein B-depleted plasma, 0.15 mM cAMP, and 2 μg/mL acyl-CoA cholesterol acyltransferase (ACAT) inhibitor in MEM-HEPES buffer at 37 °C. The extent of BODIPY cholesterol effluxed into the media was directly gauged using a spectrofluorometer plate reader (Tecan, Trading AG, Männedorf, Switzerland) at an excitation wavelength of 485 nm and an emission detection at 530 nm. CEC was computed as the ratio of effluxed BODIPY cholesterol to the initial cellular content of BODIPY cholesterol. Each analysis was conducted in triplicate, and samples exceeding a 7% variation threshold were reanalyzed.

### 2.4. Complement System Assays

The SVAR Functional Complement Assays offered under the Wieslab® brand in Sweden are utilized to evaluate the activity of the classical, alternative, and lectin pathways within the complement system. These assays employ a combination of the hemolytic assay principle for assessing complement function along with the utilization of labeled antibodies specific to the neoantigen generated as a result of complement activation. The quantity of neoantigen produced is proportional to the functional activity of the complement pathways. In these assays, microtiter strip wells are coated with activators specific to the classical, alternative, or lectin pathways. The patient’s serum is diluted using a diluent that contains a particular blocker, ensuring that only the targeted pathway is activated. During incubation of the diluted patient serum in the wells, the specific coating triggers complement activation. Subsequently, the wells are washed, and the presence of C5b-9 is detected using an alkaline phosphatase-labeled specific antibody directed against the neoantigen expressed during the formation of the membrane attack complex (MAC). Following an additional washing step, detection of the specific antibodies is achieved by incubating with an alkaline phosphatase substrate solution. The degree of complement activation is correlated with the intensity of color and quantified in terms of absorbance (optical density). The extent of MAC formation (neo-epitope) reflects the activity of the complement cascade. The test results are semiquantitatively expressed by calculating the optical density ratio between a positive control and the sample. Notably, lower levels of classical, alternative, and lectin cascade values indicate a greater activation of the respective pathway. Wieslab® has validated these functional assays by assessing their correlation and agreement with the classical CH50 and AH50 hemolytic tests (https://www.svarlifescience.com/ accessed on 15 April 2023).

### 2.5. Statistical Analysis

Demographic and clinical characteristics were presented as mean ± standard deviation (SD) or percentages for categorical variables. For continuous variables that did not follow a normal distribution, data were described as the median and interquartile range (IQR). To examine the relationship between circulating C system molecules and pathways with lipid profile-related molecules, multivariable linear regression analysis was employed. Confounding variables were selected if they demonstrated a significant association with both the exposure and outcome variables, with a p-value below 0.20. To avoid issues of collinearity, regression models excluded variables that were derived from a formula, such as LDL cholesterol, LDL:HDL ratio, non-HDL cholesterol, ApoB:ApoA1, and atherogenic index. Collinearity in the multivariable regression models was assessed using the variance inflation factor (VIF), considering a VIF value greater than 10 as indicative of collinearity. All statistical analyses were conducted with a two-sided significance level set at 5% and were performed using Stata software, version 17/SE (StataCorp., College Station, TX, USA). The *p*-values less than 0.05 were considered statistically significant.

## 3. Results

### 3.1. Demographic and Disease-Related Data of Patients with Systemic Lupus Erythematosus

Table 1 displays the demographic and disease-related characteristics of systemic lupus erythematosus (SLE) patients. The majority of the participants were women, accounting for 94% of the sample, with a mean age ± standard deviation (SD) of 50 ± 11 years. The average age of diagnosis was 35 ± 13 years, and the disease duration was 15 ± 10 years. Upon recruitment, 72% of the patients tested positive for anti-DNA antibodies, while 66% were positive for ENA (extractable nuclear antigens), with anti-SSA being the most detected antibody (38%). Twelve per cent of the patients met the criteria for associated antiphospholipid syndrome, and 35% exhibited at least one positive antiphospholipid antibody. Based on the SLE Disease Activity Index-2000 (SLEDAI-2K) score, the majority of patients fell into the categories of no activity (43%) or mild–moderate activity (62%). The median Systemic Lupus International Collaborating Clinics/American College of Rheumatology (SLICC/ACR) Damage Index (SDI) and Katz index scores were 1 (with an interquartile range—IQR of 0–2) and 2 (IQR 1–4), respectively. Seventy-five per cent of patients had an SDI score of 1 or higher, indicating some degree of damage associated with the disease. Regarding treatments at the time of assessment, half of the patients (50%) were taking glucocorticoids, with a median equivalent daily dose of prednisone at 5 mg/day (IQR 5–7.5 mg). Sixty-nine per cent of the patients were also receiving hydroxychloroquine during the study. Other less commonly used drugs included methotrexate (12%) and azathioprine (13%). Additional information related to SLE can be found in Table 1.

C assays values of the classical, alternative, and lectin pathways were 92 ± 40%, 60 (IQR 22–88)% and 12 (IQR 1–42)%, respectively; besides, single C components serum values (C1q, C2, C4, C3 (inactive zimogens) and FD), activated molecule C3a, and regulators (C1-inhibitor (C1-inh)) are shown in Table 1.

### 3.2. Disease-Related Data Association with CEC

Demographics and disease-related data association with CEC is shown in Table 1. Overall, no associations were found between disease characteristics or comorbidity and CEC. Only the use of methotrexate showed a positive significant relationship to CEC (beta coef. 3 [95%CI 0.8–4], *p* = 0.005). Similarly, a positive trend to association with CEC was observed for azathioprine use. On the contrary, age and hypertension showed a trend to be negatively associated with CEC, although, in these cases, statistical significance was not reached.

### 3.3. Full Lipid Profile Molecules Relationship to Routes and Individual Elements of the C System

**Figure 1:**
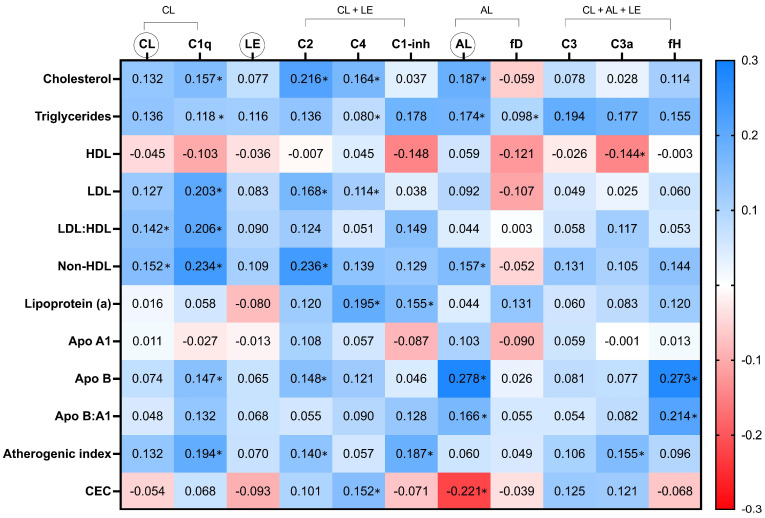
Heat map of the relationship of lipid profile molecules and complement pathways functional tests and individual elements. CL: classical, AL: alternative, LE: lectin, HDL: high-density lipoprotein cholesterol, LDL: low-density lipoprotein cholesterol, Apo: apolipoprotein, CEC: cholesterol efflux. CL, LE, and AL in circles refer to the functional tests of these cascades. Values in the cells represent Spearman’s rho coefficient (* denotes *p* value < 0.05).

Values of the lipid profile molecules assessed in the present work are shown in Figure 1 and Supplementary Table S1. The univariable relation of these lipid pattern to the cascades functional assays and individual components of the C system is exposed as Spearman’s rho correlation coefficients. In this sense, many associations were found in the univariable analysis. Regarding lipid molecules and ratios, most of the relations turned out to be positive (blue in the heatmap). The strongest ones, with a coefficient greater than 0.2, were found between C1q (classical pathway inactive zymogen) and the serum levels of LDL, LDL:HDL ratio, and non-HDL cholesterol; between C2 (common inactive zymogen of the classical and lectin pathways) and total cholesterol and non-HDL cholesterol; between the alternative pathway functional test and apolipoprotein B; and between factor H (shared regulator of the three cascades) and apolipoprotein B and ApoB:ApoA1 ratio. The only negative significant relation between the C system and lipid molecules was observed between HDL and the active protein C3a. Regarding lipid profile indexes, the atherogenic index was positively related to C1q, C2, C1-inh (regulator of both the classical and lectin pathways), and C3a. On the other hand, CEC was significantly and negatively associated with the alternative pathway functional assay and positively with C4 (common inactive zymogen of the classical and lectin cascades). Remarkably, the functional test of the lectin pathway showed no significant relation with any of the lipid pattern variables, while the alternative route functional test, together with C1q, were the C parameters more broadly related with the lipid profile (Figure 1 and Supplementary Table S1).

### 3.4. Multivariable Analysis of the Association of C System Pathways and Components with CEC

The association of C functional assays and individual C elements with CEC is represented in Table 2. We performed a multivariable analysis controlling for hypertension, Katz index, methotrexate, azathioprine (disease-related data that had a p value inferior to 0.20 in relation to CEC), statins intake, and all lipids profile molecules that were not derived from a formula (to avoid collinearity). After this adjustment, the functional assay of the alternative route showed a significant and negative relationship with CEC (beta coef. −0.02 [95%CI−0.04–−0.002], *p* = 0.030). This was also the case for C2 and C3 (inactive zymogen common to the three pathways), in which the associations were found to be positive and statistically significant (Table 2).

## 4. Discussion

The present report is an extension of a previous study in which we measured CEC in patients with SLE. In that work, we described that these patients had lower levels of CEC compared to matched controls, and that CEC was negatively and independently related to the presence of carotid plaque [6]. Our work now delves into the mechanisms that lead to this altered CEC in subjects with SLE. This study is the first in the literature to evaluate the relationship between the C system and CEC in patients in this autoimmune disease. According to our results, they could be related. In this sense, the disruption of the C system that occurs in patients with SLE may participate in the mechanisms that lead to the alteration of CEC observed in these patients.

Serum HDL cholesterol levels are inversely associated with CV disease events. It is the functional properties of HDL, in particular its reverse cholesterol transport capacity, and not simply its serum concentration, that are believed to be the key protective mechanism mediating its beneficial effect. The potential value of assessing macrophage CEC was evaluated in a study of 2924 individuals free from CV disease [2]. In this work, the primary endpoint was defined as a first nonfatal myocardial infarction, nonfatal stroke, or coronary revascularization or death from CV causes. After a median follow-up period of 9.4 years, there was a strong inverse relationship between cholesterol efflux capacity and the primary endpoint (adjusted hazard ratio (HR) 0.33, 95% CI 0.19–0.55) comparing the highest quartile with the lowest. Baseline HDL cholesterol was not associated with cardiovascular events in this study. Similarly, a recent meta-analysis has shown an inverse relationship between CEC levels and the risk of adverse CV events or atherosclerotic CV disease [13]. Moreover, CEC has also been evaluated as a CV disease risk predictor. In one study it improved risk prediction beyond that of coronary artery calcification, family history, and high-sensitivity C-reactive protein [14]. This seems to be the case not only in the general population but also in immune-mediated inflammatory diseases [15,16,17,18,19]. However, the mechanisms that link inflammation and CEC remain unknown.

C system is a complex network of proteins with a canonical role in the immune response; besides, its implication in metabolic disease processes such as atherosclerosis has been demonstrated. In this regard, it has been described that depending on the stage of C activation, C can either mitigate or promote lesion formation, and there is increasing evidence that local C expression is key to understanding atherosclerosis [20]. This has been supported by the fact that circulating C5 was significantly elevated in patients with subclinical atherosclerosis and was positively correlated with plaque volume and coronary calcification [21]. Furthermore, elevated serum C5a levels predicted future CV events in patients with symptomatic peripheral arterial disease regardless of acute phase markers [22]. Similarly, hyperactive terminal pathway activation as judged by serum C5b-9 complex levels was found to be associated with carotid plaque instability and acute ischemic stroke outcomes [23].

Regarding lipid profile molecules, a relationship between the C system and the metabolism and/or function of circulating lipoproteins has been suggested. For example, in a random population of 1068 subjects, circulating C3 levels were positively correlated with conventional CV risk factors, such as LDL cholesterol and triglycerides [24]. In the Cohort on Diabetes and Atherosclerosis Maastricht (CODAM), studies on plasma C concentrations (C3, properdin, factor H, factor D, C3a, Bb) and lipoprotein subclass profile (as measured by nuclear magnetic resonance spectroscopy) were performed in 523 participants [25]. Those with higher C3 concentrations had more circulating VLDL, IDL, and LDL and small HDL [25]. All these findings were consistent with our analysis. However, none of the previously mentioned reports evaluated a complete characterization of the C system through measurement of C pathways functional tests and individual active, inactive, and regulatory components. In our work, we found that different C pathways and components were associated with many lipid profile molecules in the univariable analysis. In addition, after multivariable analysis, the levels of C2, which can be considered to belong to the classical and lectin pathways, and the levels of C3, a common element of the three pathways, presented a positive relationship with CEC. According to that, the inactive particles C2 and C3 have a beneficial relationship with higher levels of CEC. This implies that adverse CEC may be present in patients with lower C2 and C3 levels due to active disease. We also observed a negative relationship between CEC and the alternative pathway of the C. It is difficult to infer a certain expression pattern of C in its relation to CEC. C components are known to have proinflammatory functions, including chemotaxis, exudation of plasma proteins at sites of inflammation, and opsonization of infectious agents and damaged cells [26]. We believe that the fact that inflammation increases certain C particles, and that disease also causes their consumption, motivates this complex relationship.

Unlike the alternative pathway, in our work we did not find that both the classical and the lectin functional assays were associated with CEC. Of note, the lectin pathway was not related to any of the lipid profile molecules. The usual pattern of C activation in SLE involves the classical pathway. However, a percentage of patients demonstrate a predominant activation of the alternative pathway [27]. In this sense, the alternative cascade does not require antibodies or prior contact with a microbe to function and serves as an independent immune system, capable of recognizing and destroying infecting elements. Our findings are consistent with previous experimental findings in which the alternative C pathway was causally involved in lipid metabolism. For instance, mice that are deficient in C3 showed delayed postprandial triglyceride clearance and increased fasting and postprandial free fatty acids levels [28]. In line with this, mice that lack other components of the alternative pathway, such as factor B or properdin, also showed delayed postprandial lipid clearance and altered systemic lipid levels [29,30]. Our results are also supported by the CODAM study in which independent associations were observed for most alternative pathway C components and HDL subclasses and enrichment in triglyceride [25].

A potential limitation of our study is its cross-sectional design, and that we specifically focused on patients with SLE and, therefore, did not recruit controls. We also acknowledge that most patients had low disease activity. However, given that disease activity did not show a relationship with CEC, it does not seem that SLE activity status had influenced the association between the C system and CEC. However, our study has the strength that it involves a large series of patients, with different disease patterns, and that we have analyzed a complete profile of lipid molecules.

## 5. Conclusions

Our results reinforce the claim of a link between CEC and C in patients with SLE. Non-canonical C system routes, other than classical pathway activation, might be implied. More studies are needed to better elucidate this complex relationship.

## Figures and Tables

**Table 1:** Demographics and disease-related data of SLE patients and their association with cholesterol efflux capacity.

			SLE (n = 207)		
				CEC%	
				β Coef. (95%CI)	*p*
Age, years			50 ± 11	−0.04 (−0.09–0.02)	0.19
Female			195 (94)	−0.2 (−3–3)	0.91
***SLE-related data***					
Age at diagnosis, years			35 ± 13	−0.01 (−0.06–0.03)	0.57
Disease duration, years			15 ± 10	−0.02 (−0.09–0.04)	0.46
Antiphospholipid syndrome			24 (12)	0.02 (−2–2)	0.87
*Auto-antibody profile*					
	Anti-DNA positive		110 (72)	−0.8 (−2–0.7)	0.29
	ENA positive		123 (66)	0.2 (−1–2)	0.77
		Anti-Sm	22 (12)	−1 (−3–0.8)	0.24
		Anti-ribosome	10 (10)	1 (−2–5)	0.44
		Anti-nucleosome	15 (15)	1 (−2–4)	0.42
		Anti-histone	9 (9)	1 (−2–5)	0.41
		Anti-RNP	51 (29)	−0.6 (2–0.9)	0.44
		Anti-SSA	40 (38)	0.3 (−2–3)	0.77
		Anti-SSB	4 (4)	0.7 (−10–11)	0.90
	Antiphospholipid antibodies		31 (35)	0.02 (−2–2)	0.87
*Disease scores*					
	Median SLEDAI-2K		0 (0−2)	−0.02 (−0.2–0.2)	0.84
	*SLEDAI-2K categories*				
		No activity	85 (43)	-	-
		Mild activity	78 (40)	−1 (−2–0.4)	0.17
		Moderate activity	24 (12)	−0.5 (−2–1)	0.62
		High or very high activity	9 (5)		
	Median SDI		1 (0–2)	0.03 (−0.3–0.4)	0.85
	SDI ≥ 1		156 (75)	0.3 (−1–2)	0.70
	Katz index		2 (1–4)	0.2 (−0.1–0.5)	0.28
	Katz ≥ 3		86 (42)	1 (−0.3–2)	0.12
***Comorbidity***					
	Smoking		48 (23)	−0.05 (−1–1)	0.94
	Diabetes		11 (5)	−0.4 (−3–2)	0.76
	Hypertension		85 (41)	−0.9 (−2−0.4)	0.17
	Obesity		58 (28)	−0.4 (−2–1)	0.60
	Body mass index, kg/m^2^		27 ± 6	0.04 (−0.07–0.2)	0.45
	Abdominal circumference, cm		93 ± 14	0.02 (−0.02–0.07)	0.33
***Treatment at the time of the visit***					
	Statins		54 (26)	−0.2 (−2–1)	0.79
	Aspirin		55 (27)	−0.2 (−2–1)	0.82
	Antihypertensive treatment		78 (38)	−0.5 (−2–0.7)	0.41
	Glucocorticoids		102 (50)	0.7 (−0.5–2)	0.25
	Prednisone equivalent daily dose, mg		5 (5−7.5)	−0.09 (−0.4–0.2)	0.52
	Antimalarials drugs		141 (69)	0.1 (−1–1)	0.83
	Methotrexate		24 (12)	**3 (0.8**–**4)**	**0.005**
	Azathioprine		27 (13)	1 (−0.6–3)	0.19
	Mycophenolate mofetil		17 (8)	0.2 (−2–2)	0.86
	Belimumab		3 (1)	0.4 (−4–5)	0.86
	Rituximab		6 (3)	−1 (−5–2)	0.55
***Complement system parameters***					
*Functional complement assays, %*					
	Classical pathway		92 ± 40	−0.007 (−0.02–0.009)	0.39
	Lectin pathway		12 (1–42)	−0.008 (−0.02–0.007)	0.28
	Alternative pathway		60 (22–88)	−**0.03 (**−**0.04**–**0.01)**	**0.001**
*Individual complement components*					
	C1q, mg/dL		35 ± 11	0.004 (−0.02–0.08)	0.26
	C2, mg/dL		2.6 ± 1.3	**0.5 (0.02**–**1)**	**0.043**
	C4, mg/dL		21 ± 12	0.04 (−0.01–0.09)	0.15
	Factor D, ng/mL		2749 ± 1700	−0.0001 (−0.0006–0.0003)	0.55
	C3, mg/dL		134 ± 42	0.01 (−0.0009–0.03)	0.065
	C3a, mg/dL		38 ± 11	0.03 (−0.03–0.09)	0.28
	C1 inhibitor, mg/dL		31 ± 10	−0.02 (−0.08–0.05)	0.59
	Factor H, ng/mL × 10^−3^		448 (302–745)	−0.0002 (−0.0009–0.0004)	0.45

Data represent mean ± SD or median (interquartile range) for quantitative variables, and frequency (percentage) for categorical variables. Significant *p* values are depicted in bold. In the univariable regression analysis, cholesterol efflux capacity (CEC) is the dependent variable. For categorized SLEDAI-2K ordinal variable, no activity category is taken as the reference level. SLEDAI-2K categories were defined as: 0, no activity; 1–5 mild; 6–10 moderate; >10 high activity. Aspirin use defined as 100–150 mg daily intake of acetylsalicylic acid. CEC: cholesterol efflux capacity. ENA: extractable nuclear antibodies. SLEDAI-2K: Systemic Lupus Erythematosus Disease Activity Index. SDI: Systemic Lupus International Collaborating Clinics/American College of Rheumatology Damage Index.

**Table 2:** Multivariable analysis of the association of complement system pathways and components with cholesterol efflux capacity.

		β Coef. (95%CI), *p*			
		CEC%			
		Univariable		Multivariable	
*Classical pathway*					
	Functional assay, %	−0.007 (−0.02–0.009)	0.39		
	C1q, mg/dL	0.004 (−0.02–0.08)	0.26		
*Lectin pathway*					
	Functional assay, %	−0.008 (−0.02–0.007)	0.28		
*Common elements of the classic and lectin pathways*					
	C2, mg/dL	**0.5 (0.02**–**1)**	**0.043**	**0.5 (0.005**–**1)**	**0.048**
	C4, mg/dL	0.04 (−0.01–0.09)	0.15	0.05 (−0.01–0.1)	0.11
	C1 inhibitor, mg/dL	−0.02 (−0.08–0.05)	0.59		
*Alternative pathway*					
	Functional assay, %	−**0.03 (**–**0.04**–−**0.01)**	**0.001**	−**0.02 (**–**0.04**–−**0.002)**	**0.030**
	Factor D, ng/mL	−0.0001 (−0.0006–0.0003)	0.55		
*Common elements of the three pathways*					
	C3, mg/dL	0.01 (−0.0009–0.03)	0.065	**0.02 (0.005**–**0.04)**	**0.009**
	C3a, mg/dL	0.03 (−0.03–0.09)	0.28		
	Factor H, ng/mL × 10^−3^	−0.0002 (−0.0009–0.0004)	0.45		

Complement routes and elements are considered the independent variable. Multivariable analysis is adjusted for hypertension, Katz index score ≥ 3, methotrexate, azathioprine, statins intake and total cholesterol, HDL, lipoprotein (a), triglycerides and apolipoproteins A1 and B serum levels.

## Data Availability

The data sets used and/or analyzed in the present study are available from the corresponding author upon request.

## Supplementary Materials

The following supporting information can be downloaded at: https://www.mdpi.com/article/10.3390/jcm12165405/s1, Table S1: Full lipid profile molecules relation to routes and individual elements of the complement system.
