# Supplementary material for: HDL Cholesterol Efflux and the Complement System Are Linked in Systemic Lupus Erythematosus
Source: J Clin Med. 2023 Aug 20;12(16):5405. doi: 10.3390/jcm12165405 (PMC10455830; doi:10.3390/jcm12165405)
Supplement: Supplementary file 1 [file jcm-12-05405-s001.zip › jcm-2541575-supplementary.pdf]

Table S1. Full lipid profile molecules relation to routes and individual elements of the complement system

		Spearman's rho correlation coefficient, p										
		Classical route		Lectin route	Classical and Lectin routes common elements			Alternative route		Common elements of the three routes		
		CL	C1q	LE	C2	C4	C1inh	AL	fD	C3	C3a	fH
Cholesterol, mg/dl	199 ± 38	0.1321	**0.1567**	0.0766	**0.2155**	**0.1638**	0.0372	**0.1868**	-0.0594	0.0778	0.0283	0.1142
		0.061	**0.028**	0.28	**0.002**	**0.026**	0.63	**0.008**	0.48	0.29	0.69	0.17
Triglycerides, mg/dl	128 ± 78	0.1355	**0.1182**	0.1159	0.1362	**0.0795**	0.1783	**0.1742**	**0.0979**	0.1936	0.1765	0.1551
		0.055	**0.013**	0.10	0.097	**0.022**	0.053	**0.008**	**0.013**	0.28	0.24	0.063
HDL, mg/dl	63 ± 21	-0.0449	-0.1030	-0.0364	-0.0065	0.0446	-0.1476	0.0593	-0.1207	-0.0260	**-0.1441**	-0.0034
		0.53	0.15	0.61	0.93	0.55	0.059	0.40	0.15	0.72	**0.042**	0.97
LDL, mg/dl	111 ± 29	0.1270	**0.2029**	0.0832	**0.1676**	0.1142	0.0379	0.0922	-0.1071	0.0486	0.0247	0.0603
		0.072	**0.004**	0.24	**0.017**	0.12	0.63	0.19	0.20	0.51	0.73	0.47
LDL:HDL ratio	1.94 ± 0.77	**0.1419**	**0.2055**	0.0901	0.1235	0.0507	0.1493	0.0441	0.0029	0.0578	0.1168	0.0528
		**0.044**	**0.004**	0.20	0.080	0.49	0.056	0.53	0.97	0.43	0.10	0.53
Non-HDL chol., mg/dl	137 ± 34	**0.1516**	**0.2344**	0.1090	**0.2362**	0.1390	0.1288	**0.1571**	-0.0519	0.1312	0.1049	0.1436
		**0.031**	**0.001**	0.12	**0.001**	0.059	0.099	**0.026**	0.53	0.074	0.14	0.085
Lipoprotein (a), mg/dl	40 (13-117)	0.0163	0.0575	-0.0797	0.1202	**0.1947**	**0.1552**	0.0439	0.1313	0.0598	0.0828	0.1195
		0.82	0.42	0.26	0.089	**0.008**	**0.047**	0.54	0.12	0.42	0.24	0.15
Apolipoprotein A1, mg/dl	179 ± 37	0.0114	-0.0270	-0.0133	0.1076	0.0565	-0.0868	0.1029	-0.0902	0.0588	-0.0010	0.0132
		0.87	0.71	0.85	0.13	0.44	0.27	0.15	0.28	0.43	0.99	0.87
Apolipoprotein B, mg/dl	96 ± 24	0.0741	**0.1469**	0.0651	**0.1484**	0.1208	0.0459	**0.2776**	0.0264	0.0806	0.0771	**0.2728**
		0.29	**0.039**	0.36	**0.035**	0.10	0.56	**<0.001**	0.75	0.27	0.28	**0.001**
ApoB:Apo A1 ratio	0.55 ± 0.17	0.0476	0.1322	0.0683	0.0551	0.0902	0.1278	**0.1662**	0.0546	0.0543	0.0820	**0.2141**
		0.50	0.063	0.33	0.44	0.22	0.10	**0.018**	0.51	0.46	0.25	**0.010**
Atherogenic index	3.43 ± 1.10	0.1317	**0.1940**	0.0704	**0.1403**	0.0566	**0.1871**	0.0602	0.0493	0.1063	**0.1553**	0.0955
		0.062	**0.006**	0.32	**0.047**	0.44	**0.016**	0.39	0.56	0.15	**0.029**	0.25
CEC, %	8.1 ± 4.2	-0.0544	0.0675	-0.0931	0.1005	**0.1519**	-0.0707	**-0.2213**	-0.0390	0.1252	0.1205	-0.0681
		0.46	0.36	0.21	0.17	**0.047**	0.39	**0.002**	0.66	0.10	0.10	0.44

Lipid profile molecules are shown as means ± standard deviation or median (interquartile range) when data were not normally distributed. *Significant p values are depicted in bold. HDL: high density lipoprotein, LDL: low density lipoprotein, CEC: cholesterol efflux capacity, CL: classical route functional assay, LE: lectin route functional assay, AL: alternative route functional assay, fD: factor D, fH: factor.
